# Retirement migration, the ‘other’ story: caring for frail elderly British citizens in Spain

**DOI:** 10.1017/S0144686X14001342

**Published:** 2014-12-09

**Authors:** KELLY HALL, IRENE HARDILL

**Affiliations:** * Institute of Applied Social Studies, University of Birmingham, UK.; † Department of Social Sciences and Languages, Northumbria University, Newcastle upon Tyne, UK.

**Keywords:** international retirement migration, lifestyle migration, Spain, United Kingdom (UK), vulnerability, care, old age

## Abstract

Recent years have seen a growth in research on retirement/lifestyle migration to Spain, however this has tended to focus on the reasons for moving, as well as the lifestyles adopted as part of a healthy and active retirement. However, ageing in Spain can bring challenges as a person's resources for independent living diminish. This paper draws on narrative interviews with vulnerable older British people in Spain, focusing on those who have encountered a severe decline in health, are frail and in need of care. It looks at the formal and informal networks and agencies that support these individuals, in particular the resources and strategies they employ to access care. Drawing on a framework of care provision developed by Glucksmann and Lyons, four broad modes of provision for old age care used by older British people in Spain are identified: state/public, family/community, voluntary/not-for-profit and market/for-profit. The paper argues that there are language, cultural, spatial and financial barriers when accessing care in Spain as an older British citizen. It is concluded that there are some frail, vulnerable people that may fall through a support gap, whereby they are no longer the responsibility of UK welfare services, yet not fully recognised in their new country of residence, and asks if more should be done to support this population.

## Introduction

Whilst migration to rural and coastal areas within the United Kingdom (UK) remains a common choice for older adults in retirement or pre-retirement (Lowe and Speakman 2006; Stockdale 2011), the past few decades have seen a considerable increase in the number of older British people moving abroad. Early retirement and flexible retirement options mean that the move into retirement is no longer the abrupt lifecourse stage undertaken at the state pension eligibility age that it once was (Stockdale 2011). People in their fifties and older now form part of the North–South flow within Europe (Coldron and Ackers 2007; Gustafson 2001, 2008; Innes 2008; King, Warnes and Williams 2000) and Spain has become a popular retirement destination for British people (Oliver 2008; O'Reilly 2007; Hardill *et al.* 2005). International retirement migration (King, Warnes and Williams 2000) has now been conceptualised as a form of ‘lifestyle migration’ (Benson and O'Reilly 2009) which occurs as a result of a search for ‘self-fulfilment’ or the ‘good life’ (Oliver 2007). Much of the research to date has focused on understanding the motives that prompted people to move abroad as retirement approaches (King, Warnes and Williams 2000), as well as the lifestyles adopted and social networks developed as part of a healthy retirement in Spain (Casado-Diaz 2009; Gustavson 2009). As such, most studies have been framed within the context of the ‘third age’ (Laslett 1991), in particular around the notion of ‘active ageing’ focusing on those who are visible and active within Spanish and/or British community life in Spain. This includes Caroline Oliver's seminal research on retired migrants in Spain (2004, 2008, 2010, 2011), which looked at issues around ageing and focused primarily on ‘positive ageing’ and retired migrant's conceptions of place, community and identity. She does also recognise the limits of old age for these migrants, including bodily decline, yet only briefly considers aspects of obtaining care in Spain.

Subsequently, research has largely ignored the increasing number of British people in Spain who are in the ‘fourth age’ of life (Laslett 1991) and now require additional support and care as a result of declining health and frailty. This paper, therefore, focuses on this vulnerable group who are less visible within social spaces and explores the lived experiences of accessing care as an older British person in Spain. It includes both those who reached the fourth age whilst living in Spain, as well as those who migrated in the fourth age of life. It looks at the formal and informal support networks and agencies in both Spain and the UK that support these individuals. It draws on a framework of care provision developed by Glucksmann and Lyons (2006) to identify four broad modes of provision used by older British people to access care in Spain: state/public, family/community, voluntary/not-for-profit and market/for-profit. This paper begins by providing an overview of the British community in Spain, followed by a discussion of the concepts of ‘vulnerability’ and ‘care’, as well as the health and care services available to older British people in Spain. It then provides an overview of the research methods employed before presenting our findings based around the lived experiences of accessing care in Spain as a vulnerable, older British citizen.

### The British community in Spain

The British community in Spain, while long established, is very diverse, socially, economically and geographically. Most reside in coastal locations (King, Warnes and Williams 2000) including the province of Alicante (Costa Blanca), followed by Malaga (Costa del Sol), the Canary Islands and the Balearic Islands (Instituto Nacional de Estadistica 2007). The exact number of older British citizens resident in Spain is, however, unclear because some do not register with Spanish authorities (La Parra and Angel Mateo 2008; Warnes 2002). Department for Work and Pensions (2011) figures indicate that there are approximately 100,000 retired British people living in Spain, however, other *de facto* figures suggest this is much higher (*e.g.* Sriskandarajah and Drew 2006). Official registration figures are also unclear due to the diversity of the British community in Spain, which includes permanent residents as well as those who spend part of the year in Spain and the rest in the UK.

The nature of ties that bind retired migrants to the UK and Spain can therefore vary substantially. Some maintain strong ties with friends, family and institutions in the UK (Oliver 2008), whilst for others these links with the UK are weak or have dissipated, especially those who have been in Spain for a considerable time (Hardill *et al.* 2005; Huber and O'Reilly 2004; Legido-Quigley and McKee 2012). The social networks of older British migrants can also be complex as they often transcend national boundaries, with networks in both Spain and the UK. As a result, British migrants in Spain have been labelled ‘transmigrants’ (O'Reilly 2007), whereby they belong to two or more countries at the same time and construct dual lives across national borders (O'Reilly 2007; Vertovec 2005, 2007). O'Reilly (2000) found that for most British migrants in Spain, Britain still remains part of who they are. Many ‘retain a strong dependence on their home society, sometimes financially (e.g. pension) and sometimes emotionally (e.g. family ties)’ and always have the ‘secure knowledge that if all else fails, they can (and do) just go home’ (O'Reilly 2000: 159).

As a result, British citizens resident in Spain are often accused of developing an ‘enclave mentality’ (Champion and King 1993: 54), establishing and maintaining links with other British residents, but rarely integrating and identifying with the host Spanish community (Ahmed 2012; Haas 2013; Legido-Quigley and McKee 2012). Previous studies have highlighted that few learn to speak Spanish to a level that enables them to interact on any more than a superficial level (Iszatt 2013; King, Warnes and Williams 2000; O'Reilly 2000). Most, however, do have active social lives through friendships with other British people and participation in British social clubs (Betty and Cahill 1999; Oliver 2008). These networks are characterised by reciprocity where ‘everybody helps each other out’ (Legido-Quigley and McKee 2012). Retired British communities in Spain are also characterised by a thriving voluntary and community sector, which provides a social life and social network for the more active members (O'Reilly 2000). It also plays a crucial role in the organisation of help to alleviate health and age-related problems for those in need (Haas 2013) and charitable organisations may even support and fund repatriation for those wishing to return to the UK (Oliver 2008). The charity Age Concern España is one such organisation that was established by the British community in Spain to provide information, advice and support in areas of Spain with large populations of older British people.

Voluntary organisations have been developed out of a growing recognition that ageing in Spain can bring particular challenges as a person's resources for independent living diminish. A study by Hardill *et al.* (2005: 775), undertaken in collaboration with Age Concern España, identified some older British migrants in Spain as being in ‘critical situations’, as a result of a radical decline in quality of life or a desperate need of additional support and/or income. Their research focused on the role of local groups and British charities in providing care and support, and called for more sustained research on the issues facing older British people in Spain. Haas (2013) also identified a growing number of migrants in Spain who are becoming frail and for whom health-related problems can become a major problem. He also cites the need for further research on this population group, arguing that the ‘situation of retired migrants in Spain who are in advanced old age is a critical and little understood issue’ (Haas 2013: 1380). This paper responds to these calls by providing a deeper understanding of the lived experiences of vulnerable older British people in Spain, focusing on the experiences of accessing care.

### Care and vulnerability in old age

In this section, we examine two key concepts that underpin this paper, care and vulnerability. Turning firstly to care, the concept of care is firmly established as an activity and set of relations lying at the intersection of the state, market and family (and voluntary and community sector) relations (Williams 2001). The idea of an ethic of care (which stands in contrast to an ethic of work) responds to the writing of feminist thinkers who emphasised care for others as meaningful and fulfilling to many women and took this as a premise to propose care, as a model, to be extended to the larger social arena (Gilligan 1982; Ruddick 1990). The ethic of care is not adopted in all care-related research, but has become one of the most popular theoretical perspectives used (Kröger 2009). A feminist ethic of care begins with a social ontology of connection: foregrounding social relationships of mutuality and trust (rather than dependence). Although individuals care and are cared for, care is a relational concept. Care ethics understands all social relations as contextual, partial, attentive, responsive and responsible, and involves values of empathy, responsiveness, attentiveness and responsibility – values most readily mobilised in our homes and communities (Lawson 2007). Care, as an ethic or moral orientation, places emphasis on the welfare of the collectivity as much as that of individuals (Sampson *et al.* 2005; Williams 2005).

The globalised nature of care has been highlighted by a number of geographers, such as Geraldine Pratt (1999) and Vicky Lawson (2007), who have argued that we need to think through the spatial extensiveness of care ethics. Care networks may therefore be transnational, spanning the boundaries of different countries. As mentioned above, this includes the care networks of older British people in Spain, whose networks usually span the UK and Spain. These networks often include both formal and informal support (Hardill *et al.* 2005).

In an Economic and Social Research Council (ESRC)-funded project, Glucksmann and Lyon examined the linkages between formal, informal, paid and unpaid care provision for older adult members of the host population in the UK, Italy, the Netherlands and Sweden (Glucksmann and Lyon 2006; Lyon and Glucksmann 2008). They identified four broad modes of provision of elder care (state/public, family/community, voluntary/not-for-profit and market/for-profit), which vary between countries. Informal care is largely provided by family members, but can include friends and neighbours, and is largely unpaid, and is fundamental to elder-care provision in Italy, the Netherlands and the UK (Lyon and Glucksmann 2008: 113). Formal care can be paid for or obtained free of charge and is provided by public or private institutions and not-for-profit organisations. In Italy (like Spain) very limited state-provided services exist to support older people, but informal market-based services are used especially in the form of individual migrant care workers to supplement family-based informal care (Glucksmann and Lyon 2006).

A second European study of elder care, the OASIS project, which was funded under the Fifth Framework Programme (1998–2002), examined host population elder care in five countries (including the UK and Spain) with different family cultures and welfare state regimes. Specifically, the project analysed the ways in which formal and informal care combined to meet the needs of older adults through a survey of urban populations aged over 25 years in the five countries exploring family values and care practices (Daatland and Lowenstein 2005). Across all five countries (including the UK) family help remains important. In Spain, the family remains the principal provider of elder care. Moreover, Spain has legal obligations for adult children towards older parents and as such has relatively low levels of social care services, including elder care (Daatland and Lowenstein 2005: 175). These cultural differences in care between Spain and UK can cause increased levels of vulnerability among frail older British people in Spain.

Despite difficulties in defining what is meant by ‘vulnerability’ (Chambers 2006), for older people vulnerability is associated with a decline in quality of life and is most frequently linked to ill health or frailty (Higgs *et al.* 2003). Grundy (2006: 107) defines vulnerable older people as ‘those whose reserve capacity falls below the threshold needed to cope successfully with the challenges that they face’. She argues that individual or environmental resources can be deployed to help overcome vulnerability and these primarily include reserves of mental and physical health, family relationships and social networks, and wealth/other material resources. It is not only these available resources that are important but a person's ability to draw upon them. Vulnerability is often shaped or exacerbated by inequalities, disempowerment or access to social protection. Grundy (2006) and Schroder-Butterfill and Marianti (2006) stress the importance not only of formal social protection (including social care) but informal social support from family and community networks, especially for older people.

Vulnerability in old age can therefore be reduced by limiting the number of challenges faced and by providing adequate physical, financial and social support (Grundy 2006). Peter Laslett's (1991) *A Fresh Map of Life*, which portrayed four distinct ‘ages’ of a person's lifecourse, argues that the onset of the ‘fourth age’ (dependence and decline in health) could be put off for as long as possible through appropriate behaviour during the third age, for example by not smoking or by having a private pension. This, therefore, highlights the importance of forward planning as a way in which vulnerability can be reduced. By ensuring financial and social support systems are in place, risks are reduced and a crisis may be averted. This is particularly important for retired migrants in Spain, and Haas (2013) cites the importance of good planning as a prerequisite for successful retirement migration.

In a recent paper, Brown (2012) examined the social policies of both New Labour and the Conservative–Liberal Democrat Coalition for vulnerable groups (including older adults) – so those groups who lack the capacity to protect themselves. She argues that the increased use of the idea of vulnerability in social policy under New Labour and the Coalition could be seen to be part of the trend towards the characterisation of welfare as a gift rather than a ‘right’ (Brown 2012: 48). Linking this argument to the care needs of vulnerable older members of the British community in Spain, we could see a reduction in responsibilities of the British state to the care needs of vulnerable British migrants. This includes those in Spain who are supported by the UK government through the exportability of the state pension, health-care entitlements and other benefits, as well as those who might return from Spain in the current climate of budget constraints and economic austerity.

### Health and social care in Spain for older British citizens

British pensioners who are registered as living in Spain are entitled to free health care through reciprocal arrangements existing between EU member states. This makes any costs of health care recoverable against the country of origin. For early retirees, automatic access to free and extended health care is not however guaranteed. By moving to Spain, British citizens also give up their rights to access health care in the UK. As a result, in order to retain access to the British National Health Service (NHS), some older migrants do not declare residency in Spain (Coldron and Ackers 2007). As a result, access to health care for older British people in Spain is varied, with some manipulating their rights to ensure the best level of cover (Coldron and Ackers 2007). Not registering in Spain can have negative implications, as non-resident EU citizens are only entitled to emergency health care and so long-term care is not available. Furthermore, non-registration can result in an under-funding of services, including Social Services, in areas with high numbers of migrants (O'Reilly 2004). It has also been noted by several Spanish authors (Huete and Mantecon 2012; Rodríguez, Fernández-Mayoralas and Rojo 2004) that an ageing population of British migrants in Spain generates health-care costs that authorities find difficult to meet. Furthermore, high levels of isolation, mental illness and alcoholism among lifestyle and retired migrants may contribute to this problem (Huete and Mantecon 2012; Rodríguez, Fernández-Mayoralas and Rojo 2004).

For those who are entitled to free health care, this is to the same level of cover as a Spanish national. Overall satisfaction with health services in Spain among British residents is high (Age Concern España 2006; Haas 2013; King, Warnes and Williams 2000; Legido-Quigley and McKee 2012), however, there are some significant cultural differences between health and care systems in Spain and the UK. For instance, in hospitals, it is customary for the patient's family to provide basic nursing care, by undertaking duties such as feeding and washing patients, whilst in the UK such duties would be performed by nursing staff (Age Concern España 2006). Furthermore, compared with the UK, there is a relatively low level of social service provision for older people in Spain (Tortosa and Granell 2002), with limited public after-care, long-term care and nursing facilities (Haas 2013). Once a patient is discharged from hospital there is a ‘virtual absence of community health services’ (King, Warnes and Williams 2000: 183). As mentioned above, the Spanish welfare state has historically relied on the informal role of families to provide care and support for its older citizens (Daatland and Lowenstein 2005; Leon 2010), with more than 65 per cent of care for older people being provided informally by family members (Costa Font and González 2008). Instead, Spanish state provisions are limited to the funding of social activities such as through the *Pensionistas* club (Oliver 2008). As a result, state provision of residential, day and domiciliary services is limited and coverage varies by region.

Since the Personal Autonomy and Dependent Care Law (39/2006) came into effect in 2007, more money is being invested to extend formal provision for those requiring long-term care, including home help, day and residential care, and support for carers (Costa-Font and González 2008; Eurofound 2009). However, social care is not universal and demand often outstrips supply with places in state homes still accommodating less than 4 per cent of the over 65 population in Spain (Costa Font and González 2008). Private care homes do exist (although limited) but estimates show they cost from €1,700 to over €3,500 a month (Iszatt 2013) and therefore may be too expensive for older migrants, especially as social assistance and means-tested benefits are not exportable from the UK. The recent economic crisis has also had a negative impact on the financial situation of UK pensioners in Spain, as unfavourable exchange rates have reduced the value of the British state pension considerably (Huete, Mantecon and Estevez 2012; Kershen 2009). Even for those who can afford to pay for nursing homes, finding one where English is spoken may be difficult (Hall 2011; Hardill *et al.* 2005).

Accessing care in Spain is therefore further compounded by language barriers, because as noted above, older British people tend not to be proficient in Spanish. Even those who have a good understanding of the Spanish language are unlikely to understand the complex medical terminology needed for a visit to the doctor or when receiving hospital treatment. As a result, some people are turning to the private sector, with the use of private health care relatively high among British people in Spain which La Parra and Angel Mateo (2008) suggest may be due to language barriers when using public health care. However, private health care can be very expensive, especially for older migrants who have complex care needs (Hardill *et al.* 2005) and can be of limited use when the need for long-term care arises (Dwyer 2001). Therefore, some older British people are returning to the UK to access support (Age Concern 2007; Hall 2011; Hardill *et al.* 2005). However, accessing health care, social care or financial support from the UK as a returning British citizen requires proof of residence which can be obtained through passing the habitual residency test (to pass this test someone must show a settled intention to stay in the UK). This can take a number of months and therefore immediate support may not be available to returning British nationals, including those who are frail and vulnerable.

In the remaining part of this paper, interview data are used to present the ways in which older British citizens living in Spain are accessing care, focusing on those who have experienced a considerable decline in health and are vulnerable. We draw on Glucksmann and Lyon's (2006) framework for understanding elder care, which identified four broad modes of provision for elder care: state/public, family/community, voluntary/not-for-profit and market/for-profit, which they applied to Italy, the Netherlands, Sweden and the UK. Whilst these modes of care provision are not unique to Spain, this paper uses this framework of care to illustrate the key health and care issues facing older British people in Spain, especially highlighting the language, cultural, spatial and financial barriers faced when accessing care services in Spain that may not be evident in the UK.

## Methods

This paper draws on data from an ESRC-funded CASE studentship (2005–2011), undertaken in collaboration with Age Concern England (now Age UK) and Age Concern España. Age Concern España was established in Spain in 1994 and provides specialist information and advice for British people (although not exclusively) over the age of 50. Subsequently, ‘older people’ are defined in this study as those over 50 years. Only those who were considered ‘vulnerable’ were asked to take part. The study draws on Grundy's (2006: 107) conceptualisation of vulnerability, ‘those whose reserve capacity falls below the threshold needed to cope successfully with the challenges that they face’, as well as Hardill *et al.*'s (2005) criteria of those in ‘critical situations’. Hardill *et al.* note that those in critical situations have often experienced a radical decline in quality of life due to a decline in health or lack of finance and require additional income or support. Therefore, interview participants were selected who currently were or recently had encountered a significant difficulty, and appeared to be in need of additional support. All of the participants had received some support from Age Concern España (ranging from advice to ongoing support) and whilst this may limit the sample to only those who have used voluntary services, recruiting respondents through Age Concern provided access to an otherwise hard-to-reach and often isolated population.

Interviews were conducted with 20 British households, which included a total of 25 older individuals (16 women and nine men). The indicative sample was weighted towards women because more Age Concern service users are female (Age Concern España internal data indicate that 55 per cent of service users are female). This may be explained by previous research (Dwyer and Hardill 2011) which suggests that men are more reluctant to engage with local support services, such as Age Concern. Household rather than individual interviews were chosen as members of a household tend to have a shared life history so activities, patterns and decisions are negotiated jointly (Allan 1980), and therefore by interviewing at a household level these complex household relationships and interactions were explored resulting in richer, more detailed and validated accounts (Valentine 1999). For 13 households, an individual was interviewed (one was married, 12 were single/widowed), in four households interviews were with married couples and three interviews were with one older person and other members of their wider family (usually daughters). The average age of all interviewees (excluding wider family) was 78.25 years. All participants lived in Spain for at least nine months of the year (with most living there all year round) and the number of years lived in Spain ranged from one to 34 years and therefore captures the problems associated with a recent move to Spain, as well as those who have ‘aged in place’.

Households were located in the Costa Blanca (eight households), Costa del Sol (seven households) and Mallorca (five households). These locations are where the largest Age Concern España organisations are based and the number of interviewees in each location largely reflects the relative size of the British community in each area (based on figures from Instituto Nacional de Estadistica 2007). The indicative sample does attempt to reflect the variability of the community in terms of household composition, social class, age, marital status and length of residency in Spain. The focus of the interviews was on ‘lived experience’: on personal accounts and narratives. The narrative approach was adopted to explore participant's understandings and interpretations of their ‘critical condition’ and vulnerability, as told from the perspective of the individuals involved (Dingwall and Murphey 2003; Lawler 2002). The narrative approach therefore used interviews to understand individual and collective life stories relating to participants' experiences, values and relationships, which included being ageing individuals and members of a community. These stories involved the person's interpretation of their social world as created through an interaction between the researcher and participant (Lawler 2002). Whilst an interview guide was used, the agenda was largely set by the participants who were encouraged to talk about those issues most important to them and as such to tell their stories. Narrative analysis was then performed on the interview data, with the purpose being to emphasise the stories that participants told. After transcribing the interviews, additional notes and a fieldwork diary were also drawn on to create ‘pen portraits’ of every household interviewed. These gave an overview of each participant's characteristics and their ‘narrative story’ using a range of basic thematic headings based on the research questions. The transcribed interviews and pen portraits were then entered into NVivo for coding and further qualitative analysis. A coding framework was devised based on both the theoretical interests guiding the research questions, as well as on the salient issues and recurring ideas that arose in the text itself (Attride-Stirling 2001). Coding was undertaken in three stages: first, open coding (developing broad initial categories); second, axial coding (analysing the relationships between codes and developing sub-categories); and third, selective coding (selecting cases and quotes to illustrate major themes) (Fielding 2008). Pseudonyms are used to protect the identity of individuals, and their location in Spain. The research was carried out to the standards set in the ESRC's Research Ethics Framework and the British Sociological Society's Statement on Ethical Practice. In accordance with these guidelines, the research was conducted with the welfare of participants in mind.

All of the respondents had experienced a decline in health and were in need of additional support. The severity of health problems did vary significantly, ranging from back problems or arthritis to those who had experienced a substantial decrease in their quality of life due to the effects of illnesses such as cancer, strokes, blindness or Parkinson's disease. Ill health brought increased vulnerability and frailty as well as mobility problems, which meant that carrying out everyday activities became difficult. The social context and amount of support received by respondents also varied considerably, with some having family close by (or whom they lived with) whilst others lived alone and had no local support. The typology presented below therefore reflects a diversity of social contexts and settings. Four types of care (drawing on Glucksmann and Lyon 2006) that older British people in Spain were found to use are now presented and critically discussed in turn: state/public, market/for-profit, voluntary/not-for-profit and family/community. These are, however, not independent categories as most respondents drew upon two or even three forms of care.

## State/public care: hospitals, social services and care homes in Spain

Health care in Spain, through reciprocal arrangements with the UK, was freely available to all respondents who were over state pension age and registered as living in Spain. As has been identified in previous literature (Age Concern España 2006; Haas 2013; King, Warnes and Williams 2000), statutory health-care services in Spain are considered very good. Interviewees in this study referred to hospitals as ‘excellent’, ‘brilliant’ and ‘superb’. However, as also noted in previous literature (Daatland and Lowenstein 2005; King, Warnes and Williams 2000), there are significant differences in care practices between Spain and the UK. The main difference is that in Spain, care is expected to be provided by the family and therefore is not widely available in the community, nursing homes or even in hospitals. In a Spanish hospital, care (*e.g.* washing, bathing and feeding) is expected to be performed by family members rather than nurses (Age Concern España 2006), as our interviewees discovered:

You have got to have someone in [hospital] with you. The nurses will not help you out at all … When [husband] was ill, the two girls [granddaughters] and [daughter] took turns to stay each night with him. You have to stay the night otherwise you don't get any help. When I wasn't there, the dinner, nobody gives them anything. And then they take it away. They don't feed you, they don't wash you … If you don't have anyone with you, you might as well die. (Wilma, 76, widowed)

Interviewees also found very little or no aftercare, especially upon leaving hospital, including for example with transport and wound dressing at home:

My mother had a stroke. They kept her in hospital for five days and there was nothing more they could do for her so they just bundled her off home and when I say bundled I mean this. So this was a great trauma, and when she did come home she suffered terrible because well, you just don't get any help. (Wilma, 76, widowed)

In England you have got the District Nurses and people like that, but they don't do that here. I am not knocking the service here, the medical service is perfect … But it's just one of those things they don't do. They rely mainly on families here to look after people. (Andrew, 81, married)

Other respondents who were unable to live independently and required 24/7 care had looked into obtaining a place in a residential or nursing home. As noted earlier (Costa Font and González 2008), nursing homes in Spain are very sparse and as such accessing them was a common problem faced by participants. One interviewee (Harry) was able to access a state-funded nursing home in Spain, however, he could speak no Spanish and staff in the home did not speak English. Language and cultural barriers were therefore significant challenges for him and as a result his quality of life in the home was poor, leaving him vulnerable, isolated and lonely:

Interviewer: Can the nurses understand you if you ask them something in English?

Harry: No, most of them, no. Just the odd one. They can't speak English.

I hate the food here, it's terrible I just do not like it … My daughter, thank God she brings in some cereal for me. I have that and a cup of tea. Then they decide what they are going to do [with me] … they leave me sitting there for a while. They usually take me out to the television room, and there is a door that leads out on to what they call the patio, they leave me by the door there … I go out when it's nice on the patio, by myself, I talk to myself. (Harry, 86, widowed)

Language barriers can also cause considerable problems in accessing other statutory health and care services as approximately two-thirds of all interview participants could speak little or no Spanish. Some found that doctors, nurses and care workers rarely spoke any English:

The [doctor] I had before was taught in England … but he will not speak English but everybody knows he could but he makes you speak Spanish. (Elsa, 78, widowed)

Whilst language and cultural barriers to care were recognised and even anticipated by some respondents (as the earlier quote from Andrew indicates), others were unaware of these differences and moved to Spain expecting the same level of care that they would receive in the UK. For example, Donald thought that if his health deteriorated he would be able to access a Spanish nursing home:

But I would think if I had any major problems, they would put me into one of these … health-care places. (Donald, 80, single)

This indicates both a significant lack of preparation and anticipation of some dimension of vulnerability by older British people when they move to Spain, with the result being a gap between expectations and reality when the need for care arises.

## Market/for-profit care: private care in Spain

The limited availability of statutory care and language/cultural barriers with accessing and using care services therefore led some interviewees to turn to the private sector. This includes private health insurance, which allowed respondents access to health care and hospitals where English is more widely used (either by the doctors or through interpreters) and care is more widely available:

You can get in trouble with languages at the ordinary hospitals I think … Most of them speak enough [English], the private doctors, some don't in which case they will pick up the phone and ask for an interpreter to come which is good. (Robert, 72, divorced)

The aftercare [in the UK] was there. I can't fault that whatsoever. Out here [in Spain] it is very different, very different … when you come out of hospital, it could be six, seven o'clock at night, there is no aftercare and to try and get a doctor to visit you is virtually impossible, unless you go private. (Audrey, 66, widowed)

Some respondents also turned to the private sector to obtain care at home. This includes Barbara who employed a private cleaner/care worker for three hours per week. The availability of English-speaking domestic workers is limited, however, as Leon (2010) notes, the market is largely ‘foreignised’. As a result, Barbara's care worker did not speak any English:

She has a girl come in three times a week for an hour who just tidies round and gives her a shower … She is Polish so her English is very, very broken … There's not really any communication at all. She does what she is meant to do and that's it. (daughter of Barbara, 93, widowed)

Barbara had a high level of care needs, however, she had a limited income so could only afford a private care worker for three hours per week. Barbara and her daughter looked into nursing home care in Spain, however, they found that state-funded residential care was not available whilst a private nursing home was too expensive, as the following interview with Barbara and her daughter, Jane, indicates:

Interviewer: Is there any possibility of keeping [Barbara] in a nursing home in Spain?

Jane: It's over €2,000 a month.

Interviewer: Have you looked into state-funded homes?

Jane: There aren't any. It's impossible. I have looked. I have looked. It was the first thing I tried. There are Spaniards queuing up for state homes so obviously they are going to give preference to a Spaniard anyway.

The high cost of private health care, especially in old age, has been noted by other commentators (Dwyer 2001) and means that it is not an option for most retirees in Spain. Whilst previous research has suggested that the majority of older British people who retire to Southern Europe are in a financially privileged position (*e.g.* Ackers and Dwyer 2004; Warnes 1992), this is not actually the case. Some commentators (Oliver 2008; King, Warnes and Williams 2000) have noted that some older British people living in Spain have limited financial resources, and are dependent on state pensions. This is supported through our research as at least seven of the households interviewed were entirely dependent on their British state pension (most of whom did not own their own homes), and the number of British citizens experiencing financial vulnerability has increased as a result of the recent economic crisis and austerity programme in Spain (Huete, Mantecon and Estevez 2012; Kershen 2009). This is likely to have had a significant effect on the extent to which they are able to pay for private care in Spain.

## Voluntary/not-for-profit care: the voluntary sector in Spain

The voluntary and community sector featured prominently in the lives and support networks of older British migrants. The role of this sector in supporting older British people in Spain has been previously noted (Haas 2013), especially in supporting those with little kin support or financial means to pay for care (Oliver 2008), and this is supported here. Charities and other non-governmental organisations, such as Age Concern España, are generally set up and run by the British community, with British (and some other English-speaking) volunteers supporting vulnerable British people with a range of activities, including accessing health, care and financial support services, translating, help with shopping and providing social support. These organisations were found to play a similar role to friends and neighbours (discussed in the next section), by providing emotional support during times of ill health or disability:

It's such a relief even just to talk to someone [from Age Concern España] and the amount of care and concern that has been shown towards us, it's absolutely amazing. (Mary, 81, married)

[Age Concern España volunteer] is such a good lady. When my husband was dying she said don't worry, any time of the day, I am here for you. I mean, she had never even met me before, let alone talked to me before. I am here, ring me up even if it is one o'clock in the morning, I will be here. And that to me is wonderful. (Amy, 51, widowed)

The voluntary and community sector was arguably able to bridge the gap between informal and formal care and support. Whilst they could not provide personal or medical care, they were able to provide up-to-date information in English on health and care services. This included supporting respondents to obtain care from Spanish social services, private care workers and care homes. Age Concern España was also able to support respondents in organising and even financing funerals:

When you have got a bill for €1,000, €1,500 for a funeral we just don't have it … so [Age Concern] have really helped, because [Age Concern España] paid for the funeral. (Wilma, 76, widowed)

Other voluntary-sector organisations accessed by respondents included the Royal British Legion and other British charities linked to HM Forces or occupations, such as the Royal Air Force or Royal Marines Benevolent Funds. These provided financial support to some respondents which was used to fund old-age care. Some also received care and support from other specialist British-based charities in Spain that provided support, information and even palliative care.

## Family/community care: friends and family in Spain and the UK

Most interview participants were able to obtain support with their health and care problems through their informal social networks, including from family, friends and neighbours (often supported by volunteers from formal organisations within the voluntary sector). Previous research (*e.g.* Allan 1989; Lynch 2007; Phillipson *et al.* 2001) has indicated how family and friends tend to play different roles, with family providing practical/personal care and support during illness and disability, whilst friends are less engaged and tend to provide emotional support or advice. This, however, assumes that family are living close by and regular physical contact is maintained. For those living in Spain, family usually live at a distance, as was the case for 15 of the households interviewed who had no family in Spain. Whilst most had family in the UK (*e.g.* grown-up children, grandchildren, siblings), physical contact with them was constrained by distance. The nature of family support for these people was therefore emotional, obtained through phone calls or occasionally Skype, supported by visits back to the UK or for those who were less mobile, family visits to Spain:

I had one of my daughters here and she was helping and another daughter came out as well. But they can't be here all the time obviously. (Andrew, 81, married)

This supports the research of Baldassar (2007), who found that migrants can exchange emotional care over a distance using the telephone and information and communication technologies (ICTs) however, personal care is much more difficult to provide. Therefore, whilst it was possible to provide emotionally involved care, it was not personal or primary care as this requires a physical presence:

I couldn't ask [daughter] to come out and help with all the personal things I had to do for [husband]. I couldn't ask a 30-year-old to come and do that. (Audrey, 66, widowed)

Different strategies to obtain locally based informal support were developed by migrants, including drawing on friends and neighbours in Spain. Friends were important to most participants, and this was especially true during times of ill health or disability. Friends provided a range of support, including emotional support during illness and following bereavement or during other times of crisis:

[When my husband was dying] my friends came and stayed with me and I stayed in the A&E [Accident and Emergency Department] and five o'clock, the next morning, he gave up the fight. (Audrey, 66, widowed)

[Husband] couldn't understand what was going on and he realised [I was having a stroke] and then [friends] came round. They called an ambulance. (Victoria, 67, married)

Support from friends also included practical help, such as with shopping, transportation to hospital, the translation of medical information and translating at medical appointments:

My two what I call my best friends, I have known them virtually from when I first came here … they would come to the hospital, one of them would speak to the doctor for me. (Audrey, 66, widowed)

The lady next door comes in and helps. She is Spanish. When she was here yesterday she told [wife] she has just moved and said you can ring me up any time in the morning … She said if anything happens again, if it is one, two, three o'clock, telephone me. (Felicity, 80, married)

Whilst the friends of most interviewees were British, this quote indicates that some had (English-speaking) Spanish friends and neighbours who they turned to for help, especially with the navigation through health and care services. Therefore support was not entirely limited to within the British community, although the vast majority was. Some British friends of interviewees did, however, return to the UK or died and as such this support was lost (as was also indicated by Oliver 2008).

Whilst the above indicates that most interviewees had no family in Spain, five households had daughters in Spain, three of whom live with them. Moving to Spain for these respondents was motivated by a desire to be near their children who already lived in Spain. Most had moved in their old age when they were already vulnerable and in need of additional care and support, which they wanted to obtain from their family in Spain. Whilst this could be conceptualised as a form of ‘lifestyle migration’ as the move is ultimately to enhance quality of life through obtaining support from family, the move is driven by the need to receive care rather than add ‘for’ ‘personal fulfilment’. Migration for these people was therefore a way in which vulnerability could be reduced. Those living with their daughters in Spain were able to receive emotional, practical and often personal care. As care needs increased, familial care became supported by formal care including paid care workers (Barbara) or care homes (Harry). A rapid decline in health led to a situation of critical need, where additional (often expensive or inappropriate) care was required. The result was that some of these respondents found that they had no option but to return to the UK for care.

## Discussion

Whilst previous research has addressed issues around ageing and accessing support as an older British person in Spain (King, Warnes and Williams 2000; Oliver 2008; O'Reilly 2000; Rodríguez, Fernández-Mayoralas and Rojo 2004), this has tended to focus on older migrants in the third age of life who are primarily happy and healthy, for whom care is not an imminent necessity. This paper has instead focused on those who are in their ‘fourth age’; those who are vulnerable, frail and for whom obtaining appropriate care is a serious concern. In doing so, we have identified a range of care strategies by drawing on the framework of Glucksmann and Lyon (2006) which identifies four care sectors: state/public care, market/for-profit care, voluntary/not-for-profit care and family/community care. Whilst we present these as four largely independent spheres of care, in fact the care strategies used by our respondents included a combination of most if not all of these spheres. Whilst all respondents used statutory health services (funded through reciprocal arrangements with the UK), only a small number were able to access state-funded care in Spain. Whilst we recognise that the UK does not provide universal and free care to its older population, our research and that of others (*e.g.* Age Concern España 2006; Costa Font and González 2008; Daatland and Lowenstein 2005; King, Warnes and Williams 2000; Leon 2010; Oliver 2008) does suggest a higher level of family care and therefore lower level of statutory care in Spain. As such, for our respondents, care in Spain was largely provided by the voluntary, informal and private sectors. We did also identify a subset of respondents whose move to Spain was initiated so that they could obtain care and support from their adult children who had already migrated to Spain.

We are not arguing that accessing care through these four sectors is unique to those living in Spain, as these care strategies are evident in most developed countries. Instead, we are suggesting that accessing care in Spain as a British national through each of these sectors provides unique challenges and issues that would not be evident if accessing care in the UK. In addition to differences in statutory services, living in Spain can limit the availability of face-to-face informal care and support from family, as the family of most older British people in Spain are located in the UK. Whilst family can provide some care at a distance, this is mostly emotional rather than physical, via the phone, internet and occasional visits. The care that may have been provided by family in the UK is often substituted by informal care and support from British friends and more formal support sought from UK charitable organisations that are active in Spain. Furthermore, the reliance on Spanish families to provide old-age care (as also noted by Oliver 2008) means that statutory provision of care is lower than in the UK. The British community in Spain has therefore responded to this by creating a thriving voluntary and community sector which plays an active role in supporting members of the community who are vulnerable and in need (Haas 2013), especially when it comes to care and support in old age.

Our findings suggest that older British people in Spain retain a strong dependence on the UK and this is particularly the case when it comes to care. The main sources of support are from British friends in Spain, family living in the UK (or Spain), and UK-based voluntary and community organisations active in Spain. We therefore support the idea that retired British migrants in Spain are ‘transmigrants’, whereby they belong to two or more countries at the same time and construct dual lives across national borders (O'Reilly 2007; Vertovec 2005, 2007). However, the extent to which these migrants fully integrate in Spain is debatable and therefore raises the question of whether they are fully transnational. Most speak very little if any Spanish, have few or no Spanish friends, and many turn first to the British community in Spain and then to the UK when additional support or care is needed.

We also found that there is a strong tendency to return to the UK if things go wrong and that care featured highly on the reasons for returning (supporting previous research by Ackers and Dwyer 2004). This included seven of the 20 households interviewed who were planning a return move to the UK (either immediately or within the following year). Whilst some of these return moves were planned, the majority were necessity-driven as a result of a crisis or rapid decline in health. As Age Concern (2007) has previously reported, we also found older migrants being forced to return to the UK when they can no longer live independently and there are no support systems (including care) in the host country. Support systems include statutory services, however, as this paper has identified, support frequently includes more informal structures, including friends and support provided via voluntary organisations in Spain. This support declined for some when British friends in Spain died or returned to the UK. In addition, most of the returnees in this study had little contact with either the Spanish or expatriate British communities in Spain, and as previously suggested by Legido-Quigley and McKee (2012), those who are not active members of the expatriate community can face major difficulties and they often have no option but to return home, or go into a residential home (as illustrated here through the case of Harry). Returning to the UK can, however, incur further challenges as the habitual residency test must be passed before any support can be obtained, and even then social care (especially residential care), which is in short supply, may not be immediately available.

This suggests that older migrants moving to Spain may not be putting support plans in place for when their health declines and they become frail. As noted earlier, vulnerability occurs when people are unable to cope with the challenges they face, which includes access to formal and informal social protection and support. Vulnerability can therefore be reduced by anticipating the challenges associated with old age, including the need for additional care and support. This suggests a lack of preparation and forward planning by older British migrants before they move, which can be further exacerbated by the language, culture and other barriers that make integration and settlement abroad challenging. Discourses on retirement migration recognise the importance of good planning as a prerequisite for successful retirement migration (Haas 2013) and our research indicates the difficulties that can occur as a result of poor preparation and forethought, especially when it comes to old-age care.

## Conclusion

This paper has reported research on data collected from older British people in Spain and identifies the care strategies used by those who are vulnerable and in need of additional support. As has been previously suggested by Hardill *et al.* (2005), our research indicates that some members of the older British community in Spain are falling through a support gap, whereby they are no longer the responsibility of UK welfare services, yet not fully recognised in their new country of residence. There have been arguments for more to be done to support British people living overseas (Sriskandarajah and Drew 2006), especially those on a limited or fixed income. This has led to UK-based institutions, such as charities, playing an important role in the provision of support, especially at times of crisis. The British government appears to be recognising the difficulties facing older British people in Spain, as in addition to providing information on moving abroad, the British Consulates in Spain have in recent years provided support and liaison officers and organised roadshows on pensions, benefits and health care for those living in Spain (personal communication with British Consulate in Mallorca, 20 March 2006). However, the UK government could do more to support older British citizens living overseas, including through additional financial support, *e.g.* the exportability of Pension Credit, which would enable people to purchase care and as such potentially prevent a return move to the UK. In addition, social policy in Spain should also recognise this vulnerable population and their care needs, which may be different to older Spanish citizens due to language and cultural barriers, as well as the lack of family care resources.

Further support may be required from both countries, especially since the economic downturn of 2007–2008. Economic austerity measures introduced in Spain and the Eurozone crisis have adversely affected property prices in Spain and the value of UK state pensions received in sterling. Since the economic crisis, reports suggest that fewer older British people are moving to Spain and those already living in Spain are increasingly likely to return to the UK (Huete, Mantecon and Estevez 2012) (although this may be the result of the high numbers of people who moved to Spain in the preceding years). Nonetheless, media reports cite increases in rates of homelessness, and destitution among British people in Spain, as well as an increase in the number of return moves (Rainsford 2011; Roberts 2013). In terms of care, the Spanish crisis has resulted in budget cuts of nearly 14 per cent to health and social care services in Spain (Legido-Quigley *et al.* 2013) and a lower income among British pensioners means they will be less able to pay for private care. This may place increasing pressure on voluntary services to support older people who are facing more severe problems than before the economic crisis. Further research is needed to address the impact of the economic crisis on older British people in Spain, as this vulnerable group of migrants appear to be under-represented in both UK and Spanish policy making.

## References

[ref1] Ackers L. and Dwyer P. 2004 Fixed laws, fluid lives: the citizenship status of post-retirement migrants in the European Union. Ageing & Society, 24, 1, 451–75.

[ref2] Age Concern 2007. When the Sun Sets on Retirement Abroad. 3, Age Concern Conference, London, 17 May.

[ref3] Age Concern España 2006 *Retiring to Spain*. Mallorca Age Concern España.

[ref4] Ahmed A. 2012 Structural narrative analysis: understanding experiences of lifestyle migration through two plot typologies. Qualitative Inquiry, 19, 3, 232–43.

[ref5] Allan G. 1980 A note on interviewing spouses together. Journal of Marriage and the Family, 42, 1, 205–10.

[ref76] Allan G. 1989 Friendship; Developing a sociological perspective, Hemel Hempstead: Harvester Wheatsheaf.

[ref6] Attride-Stirling J. 2001 Thematic networks: an analytic tool for qualitative research. Qualitative Research, 1, 3, 385–405.

[ref7] Baldassar L. 2007 Transnational families and aged care: the mobility of care and the migrancy of ageing. Journal of Ethnic and Migration Studies, 33, 2, 275–97.

[ref8] Benson M. and O'Reilly K. (eds) 2009 Lifestyle Migration: Expectations, Aspirations and Experiences. Aldershot, Ashgate, UK.

[ref9] Betty C. and Cahill M. 1999 British expatriates' experience of health and social care services on the Costa del Sol. In Anthias F. and Lazaridis G. (eds), Into the Margins: Migration and Exclusion in Southern Europe. Ashgate, Aldershot, UK, 83–104.

[ref10] Brown K. 2012 Re-moralising ‘vulnerability’. People, Place and Policy, 6, 1, 41–53.

[ref11] Casado-Diaz M. 2009 Social capital in the sun: bonding and bridging social capital among British retirees In Benson M. and O'Reilly K. (eds), Lifestyle Migration: Expectations, Aspirations and Experiences. Ashgate, Aldershot, UK, 87–102.

[ref12] Chambers R. 2006 Vulnerability, coping and policy. IDS Bulletin, 37, 4, 33–40.

[ref13] Champion T. and King R. 1993 New trends in international migration in Europe. Geographical Viewpoint, 21, 45–57.

[ref14] Coldron K. and Ackers L. 2007 (Ab)Using European citizenship? EU retired migrants and the exercise of healthcare rights. Maastricht Journal of European and Comparative Law, 14, 3, 287–302.

[ref15] Costa-Font J. and González A. 2008 Long-term care reform in Spain. Eurohealth, 13, 1, 20–2.

[ref16] Daatland S. O. and Lowenstein A. 2005 Intergenerational solidarity and the family–welfare state balance. European Journal of Ageing, 2, 3, 174–82. 10.1007/s10433-005-0001-1 PMC5546341 28794730

[ref17] Department for Work and Pensions 2011 *State Pension Statistics from Time Series = MAR06* Available online at http://research.dwp.gov.uk/asd/sossp.asp [Accessed May 2011].

[ref18] Dingwall R. and Murphey E. 2003 Qualitative Methods and Health Policy Research. Aldine de Gruyter, New York.

[ref19] Dwyer P. 2001 Retired EU migrants, healthcare rights and European social citizenship. Journal of Social Welfare and Family Law, 23, 1, 311–27.

[ref20] Dwyer P. and Hardill I. 2011 Promoting social inclusion? The impact of village services on the lives of older people living in rural England. Ageing & Society, 31, 2, 243–64.

[ref21] Eurofound 2009 *Law to Support Care of Dependent People, Spain* Available online at http://www.eurofound.europa.eu/areas/labourmarket/tackling/cases/es001.htm [Accessed October 2013].

[ref22] Fielding J. 2008 Coding and managing data In Gilbert G. N. (ed.), Researching Social Life. Third edition, Sage, London, 323–52.

[ref23] Gilligan C. 1982 In a Different Voice. Harvard University Press, Cambridge, Massachusetts.

[ref24] Glucksmann M. and Lyon D. 2006 Configurations of care work: paid and unpaid elder care in Italy and the Netherlands. Sociological Research Online, 11, 2.

[ref25] Grundy E. 2006 Ageing and vulnerable elderly people: European perspectives. Ageing & Society, 26, 1, 105–34.

[ref26] Gustafson P. 2001 Retirement migration and transnational lifestyles. Ageing & Society, 21, 4, 371–94.

[ref27] Gustafson P. 2008 Transnationalism in retirement migration: the case of North European retirees in Spain. Ethnic and Racial Studies, 31, 3, 451–75.

[ref28] Gustafson P. 2009 Your home in Spain: residential strategies in international retirement migration In Benson M. and O'Reilly K. (eds), Lifestyle Migration: Expectations, Aspirations and Experience. Ashgate, Aldershot, UK, 69–86.

[ref29] Haas H. 2013 Volunteering in retirement migration: meanings and functions of charitable activities for older British residents in Spain. Ageing & Society, 33, 8, 1374–400.

[ref30] Hall K. 2011. Retirement migration, the other story: the lived experiences of vulnerable, older British migrants in Spain. PhD thesis, The Nottingham Trent University, Nottingham, UK.

[ref31] Hardill I., Spradbery A., Arnold-Boakes J. and Marrugat M. L. 2005 Severe health and social care issues among British migrants who retire to Spain. Ageing & Society, 25, 5, 769–83.

[ref32] Higgs P., Hyde M., Wiggins R. and Blane D. 2003 Researching quality of life in early old age: the importance of the sociological dimension. Social Policy and Administration, 37, 3, 239–52.

[ref33] Huber A. and O'Reilly K. 2004 The construction of Heimat under conditions of individualised modernity: Swiss and British elderly migrants in Spain. Ageing & Society, 24, 3, 327–51.

[ref34] Huete R. and Mantecon A. 2012 Residential tourism or lifestyle migration: social problems linked to the non-definition of the situation In Moufakkir O. and Burns P. (eds), Controversies in Tourism. CABI, Oxford, 160–73.

[ref35] Huete R., Mantecon A. and Estevez J. 2012 Challenges in lifestyle migration research: reflections and findings about the Spanish crisis. Mobilities, 8, 3, 331–48.

[ref36] Innes A. 2008 Growing old in Malta: experiences of British retirees. International Journal of Ageing and Later Life, 3, 2, 7–42.

[ref37] Instituto Nacional de Estadistica [National Institute of Statistics] 2007 Press release, 1 March. Available online at http://www.ine.es/&sa=X&oi=translate&resnum=1&ct=result&prev=/search%3Fq%3DInstituto%2BNacional%2Bde%2BEstadistica%26hl%3Den$ [Accessed December 2011].

[ref38] Iszatt A. 2013 Care in the community. The Olive Press (Spain), 2 October, 7, 171, 6–7.

[ref39] Kershen A. 2009 Series editor preface In Benson M. and O'Reilly K. (eds), Lifestyle Migration: Expectations, Aspirations and Experiences. Ashgate, Aldershot, UK, viii–ix.

[ref40] King R., Warnes T. and Williams A., 2000 Sunset Lives: British Retirement Migration to the Mediterranean. Berg, Oxford.

[ref41] Kröger T. 2009 Care research and disability studies: nothing in common? Critical Social Policy, 29, 3, 398–420.

[ref42] La Parra D. and Angel Mateo M. 2008 Health status and access to healthcare of British nationals living on the Costa Blanca, Spain. Ageing & Society, 28, 1, 1–18.

[ref43] Laslett P. 1991 A Fresh Map of Life: The Emergence of the Third Age. Harvard University Press, Cambridge, Massachusetts.

[ref44] Lawler S. 2002 Narrative in social research In May T. (ed.), Qualitative Research in Action. Sage, London, 242–58.

[ref45] Lawson V. 2007 Geographies of care and responsibility. Annals of the Association of American Geographers, 97, 1, 1–11.

[ref46] Legido-Quigley H. and McKee M. 2012 Health and social fields in the context of lifestyle migration. Health and Place, 18, 6, 1209–16. 22986008 10.1016/j.healthplace.2012.08.005

[ref47] Legido-Quigley H., Otero L., La Parra D., Alvarez-Dardet C., Moreno M. and McKee M. 2013 Will austerity cuts dismantle the Spanish healthcare system? BMJ, 346, f2363. 23766463 10.1136/bmj.f2363

[ref48] Leon M. 2010 Migration and care work in Spain: the domestic sector revisited. Social Policy and Society, 9, 3, 409–18.

[ref49] Lowe P. and Speakman L. (eds) 2006 The Ageing Countryside: The Growing Older Population of Rural England. Age Concern England, London.

[ref50] Lynch K. 2007 Love labour as a distinct and non-commodifiable form of care labour. Sociological Review, 55, 3, 550–70.

[ref51] Lyon D. and Glucksmann M. 2008 Comparative configurations of care work across Europe. Sociology, 42, 1, 101–18.

[ref52] Oliver C. 2004 Cultural influence in migrants’ negotiation of death: the case of retired migrants in Spain. Mortality, 9, 3, 235–54.

[ref53] Oliver C. 2007 Imagined communitas: older migrants and aspirational mobility In Amit V. (ed.), Going First Class? New Approaches to Privileged Travel and Movement. Berghahn, Oxford, 126–43.

[ref54] Oliver C. 2008 Retirement Migration: Paradoxes of Ageing. Routledge, London.

[ref55] Oliver C. 2010 Between time not there and time not theirs: temporality in retirement migration to Spain. Journal of Aging, Humanities and the Arts, 4, 2, 110–18.

[ref56] Oliver C. 2011 Pastures new or old? Migration, narrative and change. Anthropologica, 53, 1, 67–77.

[ref57] O'Reilly K. 2000 The British on the Costa del Sol: Transnational Identities and Local Communities. Routledge, London.

[ref58] O'Reilly K. 2004 *The Extent and Nature of European Migrants in Spanish Society: With Special Reference to the British Case* ESRC Award Number R000223944. Available online at http://www.esrcsocietytoday.ac.uk/ESRCInfoCentre/ViewAwardPage.aspx?AwardId=2030 [Accessed May 2011].

[ref59] O'Reilly K. 2007 Intra-European migration and the mobility-enclosure dialectic. Sociology, 41, 2, 277–93.

[ref60] Phillipson C., Bernard M., Phillips J. and Ogg J. 2001 The Family and Community Life of Older People: Social Networks and Social Support in Three Urban Areas. Routledge, London.

[ref61] Pratt G. 1999 From registered nurse to registered nanny: discursive geographies of Filipina domestic workers in Vancouver, B.C. Economic Geography, 75, 3, 215–36.

[ref62] Rainsford S. 2011 UK retirement dreams going sour in Spain, charities say. *BBC News*, 21 March.

[ref63] Roberts E. 2013 The expat boom turns into a boomerang. *The Telegraph*, 28 May.

[ref64] Rodríguez V., Fernández-Mayoralas G. and Rojo F. 2004 International retirement migration: retired Europeans living on the Costa del Sol, Spain. Population Review, 43, 1, 1–36.

[ref65] Ruddick S. 1990 Maternal Thinking: Towards a Politics of Peace. Women's Press, London.

[ref66] Sampson R. J., McAdam D., MacIndoe H. and Weffer-Elizondo S. 2005 Civil society reconsidered: the durable nature and community structure of collective civil action. American Journal of Sociology, 111, 3, 673–714.

[ref67] Schroder-Butterfill E. and Marianti R. 2006 A framework for understanding old age vulnerabilities. Ageing & Society, 26, 1, 9–35. 23750062 10.1017/S0144686X05004423 PMC3672844

[ref68] Sriskandarajah D. and Drew C. 2006 Brits Abroad: Mapping the Scale and Nature of British Emigration. Institute for Public Policy Research, London.

[ref69] Stockdale A. 2011 A review of demographic ageing in the UK: opportunities for rural research population. Space and Place, 17, 3, 204–11.

[ref70] Tortosa M. A. and Granell R. 2002 Nursing home vouchers in Spain: the Valencian experience. Ageing & Society, 22, 6, 669–87.

[ref71] Valentine G. 1999 Doing household research: interviewing couples together and apart. Area, 31, 1, 67–74.

[ref72] Vertovec S. 2005 The Political Importance of Diasporas. Washington DC, Migration Policy Institute.

[ref73] Vertovec S. 2007 Migrant transnationalism and modes of transformation, In Portes A. (ed.), Re-thinking Migration: New Theoretical and Empirical Perspectives. Berghan, London, 149–79.

[ref74] Warnes A. 2002 The challenge of intra-union and in-migration to ‘social Europe’. Journal of Ethnic and Migration Studies, 28, 1, 135–52.

[ref75] Williams F. 2005 A good enough life: developing a political ethic of care. Surroundings, 30, summer, 17–32.

